# The Key Importance of Screening Underprivileged People in Order to Achieve Global Hepatitis Virus Elimination Targets

**DOI:** 10.3390/v17020265

**Published:** 2025-02-14

**Authors:** Laura Gragnani, Monica Monti, Irene De Giorgi, Anna Linda Zignego

**Affiliations:** 1 Department of Translational Research and New Medical and Surgical Technologies, University of Pisa, 56126 Pisa, Italy; laura.gragnani@unipi.it (L.G.); i.degiorgi2@studenti.unipi.it (I.D.G.); 2 Department of Experimental and Clinical Medicine, University of Florence, 50134 Firenze, Italy; monica.monti@unifi.it

**Keywords:** chronic hepatitis B (HBV), chronic hepatitis C (HCV), rapid screening, vulnerable populations, marginality

## Abstract

Chronic hepatitis B (HBV), alongside hepatitis D virus (HDV) super-/co-infection and chronic hepatitis C (HCV), are major contributors to cirrhosis, end-stage liver disease, hepatocellular carcinoma (HCC), and liver-related mortality. Despite significant progress in antiviral treatments and HBV vaccination, viral hepatitis remains a global health burden. Vulnerable populations, such as those experiencing homelessness, migrants, and economically disadvantaged groups, are disproportionately impacted by these infections, often facing barriers to care and exclusion from traditional health systems. This leads to undiagnosed cases and ongoing transmission, undermining global efforts to eliminate HBV and HCV by 2030, as outlined by the World Health Organization (WHO). Recent studies highlight the importance of tailored interventions to address health inequalities. For instance, on-site community-based screening initiatives targeting marginalized groups have shown promise, achieving higher linkage to care rates without monetary incentives. These approaches not only enhance diagnosis but also facilitate integration into healthcare systems, addressing both public health and social disparities. This review underscores the need for targeted strategies to promote the early detection and management of HBV and HCV in underserved populations. Such efforts are critical to advancing the WHO’s elimination goals, improving health outcomes, and addressing the broader social determinants of health.

## 1. Introduction

Hepatitis C (HCV) and hepatitis B (HBV) infections, along with HBV/Hepatitis Delta (HDV) co- or super-infection, have been recognized as the most common causes of the progression of many forms of chronic hepatitis to cirrhosis, the main risk factor for liver failure (ESLD; end-stage liver disease), hepatocellular carcinoma (HCC), and liver-related mortality [1,2,3,4].

According to the World Health Organization (WHO), almost 500 million people have chronic viral hepatitis, making HBV and HCV two of the top ten “infectious killers” globally [5,6,7]. Furthermore, viral hepatitis is responsible for a variety of extrahepatic pathologies which, overall, significantly increase its burden not only in terms of public health, but also in terms of the social costs of such infections [8,9]. Among the consequences of the infection, considering both its hepatic and extrahepatic aspects, are two different types of tumors: HCC and non-Hodgkin’s lymphoma.

Because chronic liver disease is asymptomatic until the late stages, estimates suggest that 40–80% of affected people are not aware that they are infected [10].

The problem appears greater in developing countries [10]; for instance, it is estimated that 5–10% of adults in East Asia and Sub-Saharan Africa may harbor a chronic HBV infection [10].

In Italy, recent (HBV) estimates by the Polaris Observatory suggest a prevalence of HBsAg positivity equal to 0.5% (0.3–0.6%) of the Italian population [11]. Regarding HCV infection, the prevalence of viremic subjects would be 1.0% (0.4–1.4%) of the population during the five-year period 2015–2022, according to Polaris Observatory data, while the ISS (Italian Istituto Superiore di Sanità) data (2019) estimated a prevalence of 0.64–0.71% of the population [12].

The availability of effective antiviral therapies for HBV and HCV and of the vaccine against HBV infection has allowed the World Health Organization (WHO) to design a strategy for the elimination of HBV and HCV viral hepatitis to be achieved by 2030 [13]. The elimination of infections involves, on one hand, a 90% decrease in new cases (incidence) and, on the other, a 65% reduction in liver-related mortality. This important accomplishment requires global action. The prevention, identification, and timely management of infections due to the major hepatitis viruses are necessary health interventions with the aim to provide healthcare that is accessible to all. However, currently, there are still several gaps in the HBV and HCV elimination policies adopted by the countries [14].

Underserved populations (i.e., homeless, underprivileged people, and migrants) still represent key targets in the micro-elimination of these viral infections. In fact, compared to the general population, people experiencing homelessness and, more generally, underprivileged individuals are at higher risk of infectious diseases and may encounter unique barriers in the receipt of healthcare as well.

More studies are needed, particularly to assess the actual scenario of hepatitis virus infections among these subpopulations [15,16].

These interventions (prevention, identification, and the timely management of infections) are essential for improving the living conditions of vulnerable people and will aid in ceasing the spread of diseases due to HBV and HCV throughout communities that, from the regional and national level, and in accordance with the WHO directives [17], are intended to experience viral infection elimination in the near future.

Usual screening strategies adopted to reach the WHO goal of HCV and HBV elimination by 2030 generally do not include those experiencing homelessness/disadvantaged people, due to problems in the linkage to care and low compliance. This is an example of health inequality with regard to these underprivileged groups. Tailored screening interventions, such as HCV and HBV testing campaigns, are essential in improving the living conditions of vulnerable populations.

In this review, we will try to report data illustrating the burden of HBV and HCV in vulnerable groups, discussing the main issues and problems in testing strategies, linkage to care, and clinical management with the help of the available data.

## 2. Materials and Methods

To develop this review, a comprehensive literature search was conducted to identify relevant studies, reports, and guidelines on chronic HBV and chronic HCV infections as well as associated health inequalities. The review focused on publications that examined the epidemiology, treatment advancements, barriers to care, and intervention strategies for underserved populations, including people experiencing homelessness, migrants, and economically disadvantaged groups. Therefore, the bibliographic databases PubMed, Scopus, and Web of Science were searched for articles published from January 2000 to December 2024. The keyword terms used in the search included “chronic hepatitis B”, “chronic hepatitis”, “migrants”, “homeless populations”, “marginalized/underprivileged/vulnerable populations”, “economically disadvantaged groups”, “health disparities”, “healthcare access” “WHO elimination goals”, and “community-based interventions”. The exclusion criteria included studies published in languages other than English, unless translations were available, and studies not providing specific data or recommendations for underserved populations.

## 3. HCV Infection Prevalences in Underprivileged and Homeless Populations: Available Data

Several studies assessing the seroprevalence of anti-HCV antibodies (anti-HCV) in underprivileged and homeless people are now available (Table 1).

Overall, similar screening approaches were used, but the results differed in terms of positivity percentage, risk factors and linkage to care strategies, as they depend on local social contexts. In other words, the characteristics of homeless populations vary across different geographical areas, which explains some discrepancies in the available reports.

Most data come from prevalence screening conducted in the USA, across different regions, cities and types of shelters. Overall, studies from North America report higher prevalence rates compared to those observed in other countries. One possible explanation for this discrepancy is the higher prevalence of intravenous drug use in the populations studied in the United States. Another contributing factor could be that many American studies are relatively older compared to those from other regions.

A screening performed from 2002 to 2003 in the Skid Row area of downtown Los Angeles, California [18], reported an anti-HCV seroprevalence in 52.5% of 198 participant homeless men who were tested at a local medical center. The very high prevalence compared to national data (1.7% in adults [41]) was attributable to the high rate of intravenous drug users (IDUs) among the tested men. Interestingly, the duration of homelessness was identified as a risk factor for HCV positivity [18].

The same research group conducted a further analysis in 2012 on 534 homeless men and women, living in the same area of Los Angeles [19]. While the anti-HCV prevalence was lower than previously reported (26.7%), drug use and chronic homelessness were confirmed as major risk factors. Indeed, 70% of HCV-positive individuals had experienced homelessness for a significant period and 77.6% were drug users [19] (Table 1).

A more general picture was achieved by including different health care clinics for the homeless [20]. In fact, eight centers were chosen from geographically diverse urban settings in order to ensure a wider territorial coverage of the USA. The total sample was 387 homeless subjects; more than 70% were males. The overall prevalence of anti-HCV positivity was 31.0%, including 70% among IDUs and 15.5% among reported non-users. This observation confirmed previous findings regarding the IDU as the first independent risk factor for HCV among US homeless people. Among IDUs, risk factors included incarceration and ≥3 years of ID use, while, among nonusers, risk factors included tattoos and imprisonment [20]. Each participant received a cash incentive [20].

The Department of Veterans Affairs (VA) is the largest provider of both human immunodeficiency virus (HIV) and HCV care in the US, providing assistance to homeless and non-homeless veterans [31]. Noska and co-workers performed an HCV prevalence analysis of the extensive VA System database, stratifying the total veteran population into homeless and non-homeless. The rate of positive viremia (HCV-RNA in the serum) was 15.3% for the homeless subgroup and 4.5% for the non-homeless subgroup [31]. As the authors stated, the size of the population of homeless veterans (242,740 homeless veterans and 5,424,685 non-homeless veterans) supports the validity of the results, which showed a much higher HCV infection prevalence in the homeless subgroup than in the non-homeless counterpart [31]. In addition, HCV prevalence is higher than what is estimated for the general US population [42].

From 2001 to 2004, Schwarz and colleagues investigated the anti-HCV seroprevalence among homeless families in the Baltimore, Maryland area, to assess positivity rates in both children and their caregivers [21]. Families were selected as one or more children living with caregivers who function as parents, in homeless shelters or transitional houses. While drug exposure was a conceivable risk factor among caregivers, the risk for children could stem from potential contact with blood of infected family members (Table 1). Notably, nearly 60% of positive caregivers were unaware of their status and 70% admitted prior ID use [21], a percentage consistent with previous analyses [19].

Ferreira et al. reported a screening program in Central Brazil that found a 2.5% HCV prevalence among 481 homeless men while the national prevalence in men is 1.15% [22]. Interestingly, ID use was reported by only 8.7% of the participants, while a higher percentage reported cocaine use (37.1%) and crack use (53.1%), which are not considered high risk behaviors for HCV infection [22] (Table 1).

In European countries, the context of social exclusion is different and the relationship between homelessness and HCV infection has dissimilar aspects.

A 2002 study in Oxford investigated HCV prevalence among the homeless, estimating it at 26.5% among 98 screened individuals. The main risk factors were past and present ID use, age over 20, and sharing injection tools. Participants received a GBP 5 shopping voucher [43].

A similar screening campaign in Lyon, France, from 2003 to 2004, tested 944 underprivileged individuals, finding a 4.7% anti-HCV Ab prevalence, with 55% confirmed as RNA-positive. However, no information on treatment outcomes was provided [32].

In Italy, no dedicated studies have assessed HCV prevalence in homeless populations. A survey at a municipal dorm in Padua found that 13.5% of 59 respondents self-reported as HCV-positive [23]. Some studies have examined related at-risk groups, including migrants, prisoners, and sex workers (Table 1). Among immigrants tested in southern Italy, HCV prevalence ranged from 1.6% to 4.5%, depending on the population and location [28,29,30]. Prison screenings revealed a 10.4% prevalence, with a significant portion of viremic cases treated successfully [15,33]. A study in Palermo found a very low HCV prevalence among sex workers, likely due to their countries of origin. This finding reinforces the understanding that sexual transmission is not a major route for the virus [26].

In a study carried out in the Tuscany region of Italy [34], we performed HBV and HCV screenings among migrants and marginal people to reveal hidden infections and link to care positive subjects (see below for more details). Regarding the methods used, all participants provided informed consent, which was available in multiple languages. Patients were tested for anti-HCV and HBsAg positivity using capillary blood sampling. Anti-HCV- and/or HBsAg-positive patients underwent second-level screenings and were treated or monitored. The screenings were conducted with an on-site strategy at charities, using finger-prick tests. Positive subjects were referred to the Interdepartmental Hepatology Center MASVE of the University of Florence or, when opportune, to the closest outpatient clinic. From January 2019 to May 2024, excluding a pause due to COVID-19, 2200 people were approached, and 1812 (82.4%) agreed to testing. A total of 52/1812 (2.9%) were anti-HCV+, with a higher proportion of Italians (*p* < 0.001) and lower education level (*p* < 0.01) compared to the anti-HCV-negative people. Intravenous drug use was an independent factor for being anti-HCV+ (*p* < 0.0001). Of the anti-HCV subjects requiring a clinical evaluation, 37.8% were linked to care and all of the 11/14 (88.6%) viremic patients were successfully treated. In conclusion, we found higher HCV positivity compared to national prevalences. Participation and linkage to care were successful. HCV risk factors could be summarized as a lifestyle characterized by extreme marginality. Similar results were also observed concerning HBV infection (see below for details) [34]. The results stress the need to implement screening of marginal groups to target residual infection “pockets”, reducing disparities in healthcare and advancing towards the WHO 2030 elimination goal.

## 4. HBV Prevalence in Underprivileged and Homeless Populations: Available Data

The prevalence of HBV markers worldwide is correlated with infection endemicity and substantially differs across geographical areas (Table 1).

In the USA, the prevalence of HBsAg-positive subjects assessed in the general population is 0.3–0.4% [44], although these data do not take into account homeless populations.

Several studies are available on HBV distribution in vulnerable populations that divide the individuals into different subgroups: homeless and non-homeless veterans [31], gay and bisexual young homeless [27], drug users [35]. The HBV prevalence was evaluated by detection of different biomarkers.

Noska and co-workers reported the presence of HBsAg in 1.8% of homeless veterans and in 1.32% of non-homeless veterans referred to the services offered by different sites of the Department of VA [31]. Of note is that the HBV positivity rate included subjects with positive HBV serological samples as well as those who reported HBV in their “Problem List” folder but did not have laboratory evidence of infection. Although the number of the screened subjects is substantial (Table 1), HBV testing was performed at a lower rate than for HCV and HIV. This is attributable to the policies of the VA, which have more treatment programs available for HCV and HIV than for HBV [31].

Considering other possible risk factors such as drug abuse and unprotected sex, a study conducted in 2013 in the Hollywood, California area analyzed the prevalence of HBV in a group of gay and bisexual homeless people, among whom 92% were currently or previously IDUs [27] (Table 1). Despite the small number of participants and the fact that the presence of anti-HBs may also include individuals without HBV infection (recovered or vaccinated individuals), such a high prevalence of HBV markers strongly suggested the lack of education concerning prevention, in a population vulnerable to infections

Another study on female sex workers, conducted between March 2001 and April 2003 in Miami, Florida, evidenced a 53.3% positivity to anti-HBV core protein (anti-HBc) [24]. All of the participants considered themselves homeless or had previously experienced homelessness (Table 1). The screening evaluated the presence of the anti-HBc, which indicates a lower prevalence of an active infection [24].

A recent analysis by Khouzam et al. [45], reporting retrospective data of a screening conducted in 2003 in the Skid Row area of downtown Los Angeles, revealed the presence of anti-HBc in 30.9% and the presence of HBsAg in 2.9% of the homeless individuals participating the study. Furthermore, the authors evidenced a coinfection in 0.5% and 0.1% of individuals with HCV and HIV, respectively. The risk was higher in those who had used IDs in the previous 12 months, had a history of opioid abuse, multiple sexual partners and recent sexual activity in exchange for cash or drugs, confirming these behaviors as major risk factors of HBV transmission.

Similar findings regarding risk behaviors were reported in a 2018 epidemiological analysis conducted in Philadelphia, Pennsylvania involving 438 syringe users [35] (Table 1). The most significant risk factors included ID (72.5%), homelessness (55.8%), a history of incarceration (87%) and non-commercial tattooing (58.8%). Furthermore, as previously noted in other studies [27], the lack of knowledge about HBV disease and prevention strategies highlighted the need to improve screening and awareness campaigns among at-risk populations [35].

As previously mentioned, the features of vulnerable populations differ depending on geographical areas.

A screening conducted in Paris, France, included homeless people along with sex workers (mostly Chinese) and IDUs [36]. The authors did not analyze results separating homeless subgroups from the others, but with 341 participants, the total rate of HBV marker (antibodies and antigens) prevalence was 6.51% [36].

A similar campaign in London was able to reach a wide range of vulnerable people [46]; 289 of the 346 participants reported previous homelessness and 53.5% were currently homeless [46]. The study aimed to improve understanding of HBV vaccination status in this setting. Findings revealed that only 52% of participants had completed the vaccination course, underscoring the urgent need for public health efforts to enhance vaccine delivery systems and address barriers to vaccination among these vulnerable populations.

The adherence to vaccination protocols is another important aspect to analyze. A Brazilian study, conducted among homeless people in Goiânia [47], showed that only 19.5% of participants presented a serological profile of prior immunization, confirming the low frequency of hepatitis B vaccination among disadvantaged populations in that region and elsewhere [47,48].

In Italy, in a study on a small population of 52 homeless individuals, mostly younger than the medium baby-boomer age (born between 1946 and 1964), commonly considered a risk factor, 26.9% were previously vaccinated for HBV [49], a very small percentage taking into account the young age of the sample.

In a recent study conducted in the Tuscany region of Italy [34], we screened migrants and marginalized individuals for HBV and HCV infections using an on-site approach at charitable organizations, incorporating finger-prick testing. The aim was to uncover hidden infections and connect individuals who tested positive to appropriate care.

From January 2019 to May 2024, excluding a pause due to the COVID-19 pandemic, 2200 individuals were approached, of whom 1812 (82.4%) consented to testing. Among these, 80 participants (4.4%) tested positive for HBsAg, with the majority being men (*p* < 0.001) and non-native Italians (*p* < 0.001) compared to those who tested negative for HBsAg. Of the HBsAg-positive individuals, 66.3% were successfully linked to care, and 90.4% were retained in care, either undergoing treatment or being monitored.

The findings highlighted that the majority of HBsAg-positive individuals were young and primarily from regions with low vaccination coverage, emphasizing geographical origin as a significant risk factor [34,46,48].

## 5. Discussion

Chronic viral hepatitis has been a significant global health concern for years. Mortality rates have been greatly influenced by the progressive decline in HCV and HBV circulation, driven by improved economic and hygienic health conditions, the introduction of the anti-HBV vaccine, and the availability of effective antiviral therapies.

With regard to HCV, even in the absence of a vaccine, direct-acting antivirals (DAAs) can achieve a cure rate of over 98% of patients [2]. These therapies, therefore, serve as the primary tool for reducing morbidity and mortality while also helping to curb the spread of infections.

For HBV infections, which may also involve hepatitis Delta virus (HDV), anti-HBV vaccination has played a crucial role in reducing the incidence of new cases [1,4]. Additionally, the development of effective antiviral drugs has significantly reduced mortality associated with chronic liver disease [1].

It is evident that the most significant advances in changing the natural history of these infections rely heavily on early identification. These infections are often asymptomatic until they reach advanced, sometimes irreversible, stages.

This highlights the need to focus on populations at high risk of being overlooked by current health measures due to extreme marginalization. Such individuals—homeless people and those experiencing severe social and housing insecurity—represent a vulnerable gap in traditional contact tracing and healthcare systems, particularly in managing widespread diseases.

This situation gives rise to two major issues: (1) inequities in access to care; (2) the persistence of hidden, uncontrolled pockets of infection.

In light of these challenges, marginalized populations are recognized as a critical barrier to achieving the WHO’s goals for eliminating viral hepatitis. Consequently, effective screening methods are urgently needed to diagnose these silent infections.

An analysis of previous efforts to reach these populations through various strategies has shown mixed results. Effectiveness often depends on local socio-economic conditions, with the most promising outcomes observed in initiatives that offered monetary incentives [18,19,43,50,51,52]. However, such policies are often impractical in most national contexts. Encouragingly, recent studies using on-site screening without monetary incentives have demonstrated highly positive results. For instance, a recent Italian study [34] targeted marginalized individuals such as homeless people, immigrants, and those facing socio-economic hardship. Free testing strategies were implemented at locations frequented by these populations, such as community meal services. Mealtimes provided an opportunity for meaningful engagement, fostering trust and enabling follow-up steps such as guiding and supporting individuals towards therapeutic centers.

Interestingly, this study found HBV and HCV prevalence rates significantly higher than recent national averages. Moreover, the linkage to care rate for those screened and subsequently treated was 70%, exceeding results from similar studies in marginalized settings, even those offering financial incentives for participation [19]. The lower linkage to care rate observed in the anti-HCV-positive individuals compared to those HBsAg-positive reflects the extreme marginalization that describes the first subset [34]. Previous studies have identified multiple barriers preventing adequate linkage to and retention in care of HCV-positive individuals, with poverty being the primary contributing factor [53]. 

In this setting, the best strategy to increase the uptake of HCV testing and linkage to HCV care is likely the reflex test for assessing viremia. One limitation of the study conducted by Monti et al. is the use of Rapid Diagnostic Tests (RDTs) [34]. While RDTs are a valuable method for assessing HBV infection by detecting HBsAg positivity, diagnosing an active HCV infection requires a second test, as RDTs only detect anti-HCV antibodies. Overall, RDTs remain highly effective tools for large-scale testing, providing quick results in a non-invasive manner and at a very affordable cost.

Overall, the results of this pioneering study highlight the need for additional, possibly broader, studies conducted in different regions using a similar approach (onsite screening without monetary compensation).

Migrants constitute a significant portion of this marginalized population. In some cases, being a foreigner can lead to the denial of rights or an inability to exercise them, including access to residency, housing, and healthcare. These barriers often exclude migrants from social and health systems. Notably, in Italy, the risk of death from infectious diseases is twice as high for foreign men compared to Italian men, highlighting stark health disparities (see https://www.ars.toscana.it/images/pubblicazioni/Collana_ARS/2024/progetto_secondi_xweb.pdf, accessed on 8 February 2025).

Overall, the results underscore the importance of implementing targeted screening programs for marginalized groups to address “pockets” of HBV and HCV infections. Such initiatives can reduce healthcare disparities and contribute significantly to achieving the WHO’s goal of viral hepatitis elimination by 2030.

This study also demonstrated that a comprehensive and coordinated approach engaging medical professionals, shelter staff, and social care workers is essential for ensuring effective healthcare delivery to vulnerable populations. Addressing their health needs holistically can help reduce disparities and improve outcomes.

Expanding screening campaigns holds great potential for identifying residual HBV and HCV infections and advancing progress towards the WHO’s 2030 elimination targets.

## 6. Conclusions

The health challenges faced by vulnerable populations, including migrants, the underprivileged, and homeless individuals, are of growing concern. Undetected, residual HBV and HCV infections within these groups contribute to ongoing transmission and exacerbate health inequities.

However, traditional screening strategies often fail to include homeless populations due to low linkage to care rates and low compliance. Developing an effective screening strategy for these groups is essential to:

- Promote health and integrate vulnerable populations into the healthcare system.
- Support the WHO’s goal of eliminating HBV and HCV as public health threats, particularly among migrants and economically disadvantaged groups.
- Conduct epidemiological studies to improve understanding of viral hepatitis in underserved populations.
- Educate individuals about prevention and treatment options for viral hepatitis.

Considering our recent findings alongside data from the literature, we can identify the key strategies to maximize adherence to screening as well as improve linkage to and retention in care. These strategies include the following:

- performing the testing directly at the charity (onsite strategy);
- using RDTs that provide a result in 10–15 min (for HCV infection assessment, the reflex test for viremia is preferable);
- making an appointment immediately after communicating the test results or, at the latest, the day after, providing easy and quick access to care;
- actively involving navigators in the screening sessions and in the further clinical path (integrated care models).

The analysis of available data strongly suggests that implementing such a strategy could uncover hidden “pockets” of HCV infection while addressing health inequalities and advancing public health goals.

## Figures and Tables

**Table 1:** Main studies concerning screening strategies used to reveal HBV/HCV infection in marginal people.

Study	Country	Sample Features	Sample Size	HCV Markers (%)	HBV Markers (%)	Risk Factors
***Studies on homeless citizens***						
Stein et al., 2004 [18]	USA	Homeless men	198	52.5 (Ab)	-	IDU; homelessness
Gelbert et al., 2012 [19]	USA	Homeless	534	26.7 (Ab)	2.9 HBsAg, 30.9 anti HBc	IDU; homelessness
Strehlow et al., 2012 [20]	USA	Homeless	387	31 (Ab)	-	IDU; tattoos; incarceration
Schwarz et al., 2003 [21]	USA	Homeless families in shelters	336 children, 168 caregivers	0 children 4.7 caregivers (Ab)	-	IDU
Pereira et al., 2013 [22]	Central Brazil	Homeless men	481	2.5 (Ab)		Age; lack of family life; IDU; number of sexual partners; STIs
Levorato et al., 2017 [23]	Italy	Homeless	59	13.5 (Ab)	-	-
Sherriff et al., 2003 [24]	UK	Homeless	98	26.5 (Ab)	-	IDU; age
Khalili et al., 2021 [25]	USA	Homeless	766	21.1 (Ab)	-	IDU
***Studies on settings with sex-related risky behaviors***						
Prestileo et al., 2013 [26]	Italy	Sex workers	239	0.4 (Ab)	-	-
Nyamathi et al., 2013 [27]	USA	Gay and bisexual young homeless	267	-	52 anti HBs	IDU
Inciardi et al., 2006 [24]	USA	Female sex workers	586	29.6 (Ab)	53.3 (anti-HBc)	IDU; age; prostitution
***Studies on migrants***						
Coppola et al., 2020 [28]	Italy	Migrants	3839	1.6 (Ab)	1.9 HBsAg	-
Cuomo et al., 2019 [29]	Italy	Migrants	304	3.3 (Ab)	12.2% HBsAg	-
Tafuri et al., 2010 [30]	Italy	Migrants	529	4.5 (Ab)	-	-
***Studies including different settings of marginalized citizens***						
Noska et al., 2017 [31]	USA	Homeless and non-homeless veterans	HCV-test: 242,740 homeless; 5,424,685 non-homeless HBV-test: 128,262 homeless; 1,499,203 non-homeless	15.3 homeless 4.5 non-homeless (HCV-RNA)	1.8 homeless 1.32 non-homeless	-
Sahajian et al., 2007 [32]	France	Underprivileged	944	4.7 (Ab)	-	-
Fiore et al., 2020 [33]	Italy	Prisoners	2376	10.4 (Ab)	-	-
Monti et al., 2025 [34]	Italy	Marginalized citizens and immigrants	1812	2.9 (Ab)	4.4 HBsAg	IDU (anti-HCV); Geographical origin (HBsAg)
Figgatt et al., 2020 [35]	USA	Drug users	337	-	1.9 HBsAg, 24.3 (anti-HBc)	IDU; homelessness; incarceration
Legoupil, 2017 [36]	France	Homeless and sex workers	341	9.76 (Ab)	6.51 HBsAg	-
Evans et al., 2018 [37]	UK	ED patients	3290	2.0 (Ab)	0.8 HBsAg	homelessness
Barror et al., 2019 [38]	Ireland, UK, Spain and Romania	Prisoners, drug users, homeless	2079	37.0 (Ab)	-	IDU
Pinazo-Bandera et al., 2024 [39]	Spain	Drug users and homeless	271	10.3 (Ab)	-	IDU; comorbidities
Segala et al., 2024 [40]	Italy	Immigrants and homeless	143	9.4 (Ab)	14.1 HBsAg	-

IDU: intravenous drug use; ED: emergency department, Ab: antibody.
